# *Bifidobacterium adolescentis*-derived hypaphorine alleviates acetaminophen hepatotoxicity by promoting hepatic *Cry1* expression

**DOI:** 10.1186/s12967-024-05312-6

**Published:** 2024-05-31

**Authors:** Ping Qin, Yanru Li, Yangjing Su, Ze Wang, Rong Wu, Xiaoqi Liang, Yunong Zeng, Peiheng Guo, Zhichao Yu, Xintao Huang, Hong Yang, Zhenhua Zeng, Xiaoshan Zhao, Shenhai Gong, Jiaochan Han, Zhongqing Chen, Wei Xiao, Ali Chen

**Affiliations:** 1 Center for Drug Research and Development, Guangdong Provincial Key Laboratory of Advanced Drug Delivery System, Guangdong Pharmaceutical University, Guangzhou, 510006 China; 2 Department of Critical Care Medicine, Nanfang Hospital, Southern Medical University, Guangzhou, 510515 China; 3 School of Traditional Chinese Medicine, Southern Medical University, Guangzhou, 510515 China; 4 School of Nursing, Southern Medical University, Guangzhou, 510515 China; 5 Department of Critical Care Medicine, The Third Affiliated Hospital of Southern Medical University, Guangzhou, 510665 China; 6 The First School of Clinical Medicine, Southern Medical University, Guangzhou, 510515 China; 7 Guangzhou Women and Children’s Medical Center, Guangzhou Medical University, Guangzhou, 510623 China; 8 Key Laboratory of Glucolipid Metabolic Disorder, Ministry of Education, Guangdong Pharmaceutical University, Guangzhou, 510006 China

**Keywords:** Acetaminophen, AILI, Gut microbiota, *Bifidobacterium adolescentis*, Hypaphorine, Oxidative stress

## Abstract

Acetaminophen (APAP)-induced liver injury (AILI) is a pressing public health concern. Although evidence suggests that *Bifidobacterium adolescentis* (*B. adolescentis*) can be used to treat liver disease, it is unclear if it can prevent AILI. In this report, we prove that *B. adolescentis* significantly attenuated AILI in mice, as demonstrated through biochemical analysis, histopathology, and enzyme-linked immunosorbent assays. Based on untargeted metabolomics and in vitro cultures, we found that *B. adolescentis* generates microbial metabolite hypaphorine. Functionally, hypaphorine inhibits the inflammatory response and hepatic oxidative stress to alleviate AILI in mice. Transcriptomic analysis indicates that *Cry1* expression is increased in APAP-treated mice after hypaphorine treatment. Overexpression of *Cry1* by its stabilizer KL001 effectively mitigates liver damage arising from oxidative stress in APAP-treated mice. Using the gene expression omnibus (GEO) database, we verified that *Cry1* gene expression was also decreased in patients with APAP-induced acute liver failure. In conclusion, this study demonstrates that *B. adolescentis* inhibits APAP-induced liver injury by generating hypaphorine, which subsequently upregulates *Cry1* to decrease inflammation and oxidative stress.

**Supplementary Information:**

The online version contains supplementary material available at 10.1186/s12967-024-05312-6.

## Introduction

Drug-induced liver injury (DILI) occurs when drugs taken incorrectly cause liver damage, characterized by high mortality [1, 2]. According to the U.S. Acute Liver Failure (ALF) Study Group, the prevalence of APAP overdose among ALF cases has been relatively stable over the past decade and APAP is the most important etiology of ALF [3]. ALF induced by APAP overdose causes nearly 500 deaths annually in the U.S. [4]. At safe therapeutic doses, 90% of APAP is converted into glucuronide and sulfate metabolites and then excreted in the urine. The remaining APAP is converted into toxic *N*-acetyl-*p*-benzoquinone imine (NAPQI) by hepatic cytochrome p450 2E1 (CYP2E1) [5]. During APAP overdose, hepatic mitochondrial glutathione (GSH) stores are rapidly depleted by excessive cumulative NAPQI. The residual NAPQI can bind mitochondrial proteins to form toxic APAP protein adducts, ultimately inducing oxidative stress [6]. *N*-Acetylcysteine (NAC) is a widely accepted APAP-induced hepatotoxicity treatment, but it has limited therapeutic benefits and dose-dependent side effects [7]. Therefore, new treatment options are urgently needed to address the liver injury caused by APAP.

The progression of liver disease is closely associated with intestinal microflora disorders [8, 9]. Translation of gut microbiota from ALF mice could aggravate liver damage in APAP-treated recipient mice [10]. Conversely, some studies indicate that probiotics reverse APAP-induced liver damage. We recently found that β-galactosidase encoded by *Lactobacillus vaginalis* releases daidzein from food and protects against APAP-induced hepatotoxicity [11]. Moreover, *Akkermansia muciniphila* activates the phosphatidylinositol 3-kinase (PI3K)/Akt signaling pathway to alleviate AILI [12]. Sedanolide as a microbe metabolite of *Bifidobacterium longum R0175* exerts antioxidant effects in APAP-treated mice [13]. *B. adolescentis* is a well-recognized probiotic, as evidenced by its ability to maintain intestinal barrier function and decrease pro-inflammatory cytokine expression after gut colonization [14, 15]. Furthermore, *B. adolescentis* attenuates steatohepatitis by preventing lipid peroxidation and NF-κB activation and produces short-chain fatty acids to alleviate non-alcoholic fatty liver disease [16, 17]. However, it is unclear whether *B. adolescentis* can reproduce hepatoprotective activities in APAP-treated mice.

As an indole alkaloid, hypaphorine is primarily enriched in medicinal plants such as *Crocus sativus* and *Caragana korshinskii Kom*. [18, 19]. Functionally, hypaphorine prevents RANKL-induced osteoclastogenesis by reducing mitogen-activated protein kinase (MAPK) and NF-κB activation [20]. In addition, hypaphorine inhibits the p38/JNK signaling pathway and reduces the pro-inflammatory response, thereby alleviating sepsis-induced acute lung injury [21]. Hypaphorine effectively alleviated tetra chloromethane-induced acute liver injury by inhibiting the inflammatory response [22]. Microbiological culture techniques suggest that hypaphorine was isolated from the fungus *Pisolithus tinctorius* [23, 24], but no relevant report indicates that it is produced by bacteria.

Cryptochrome 1 (*Cry1*) is a crucial transcriptional inhibitor in the negative feedback loop of the core molecular clock that is correlated with the length of the circadian period [25, 26]. The upregulation of *Cry1* can modulate the Toll-like receptor (TLR)/NF-κB pathway, mitigating atherosclerosis progression [27]. Overexpression of *Cry1* inhibits hepatic gluconeogenesis through the SREBP1c-*CRY1* signaling pathway. *Cry1* is present in the mitochondria and plays a crucial role in regulating the oxidative stress response [28]. In our previous study, we found that APAP hepatotoxicity exhibits circadian rhythmicity. 1-phenyl-1,2-propanedione, a metabolite produced by gut microbiota, alleviates APAP-induced rhythmic hepatotoxicity [29]. Altering the circadian rhythm may influence APAP metabolism and intestinal permeability, exacerbating AILI [30]. Thus, *Cry1* is a key gene in the circadian rhythmicity pathway that should be further explored in relation to AILI.

Herein, we aimed to evaluate the potential beneficial effects of *B. adolescentis* on APAP-induced liver injury, which is attributed to increased hypaphorine generation leading to upregulated *Cry1* gene expression. This study reveals a potential therapeutic strategy for DILI.

## Results

### *B. adolescentis* attenuates APAP-induced hepatotoxicity in mice

**Fig. 1:**
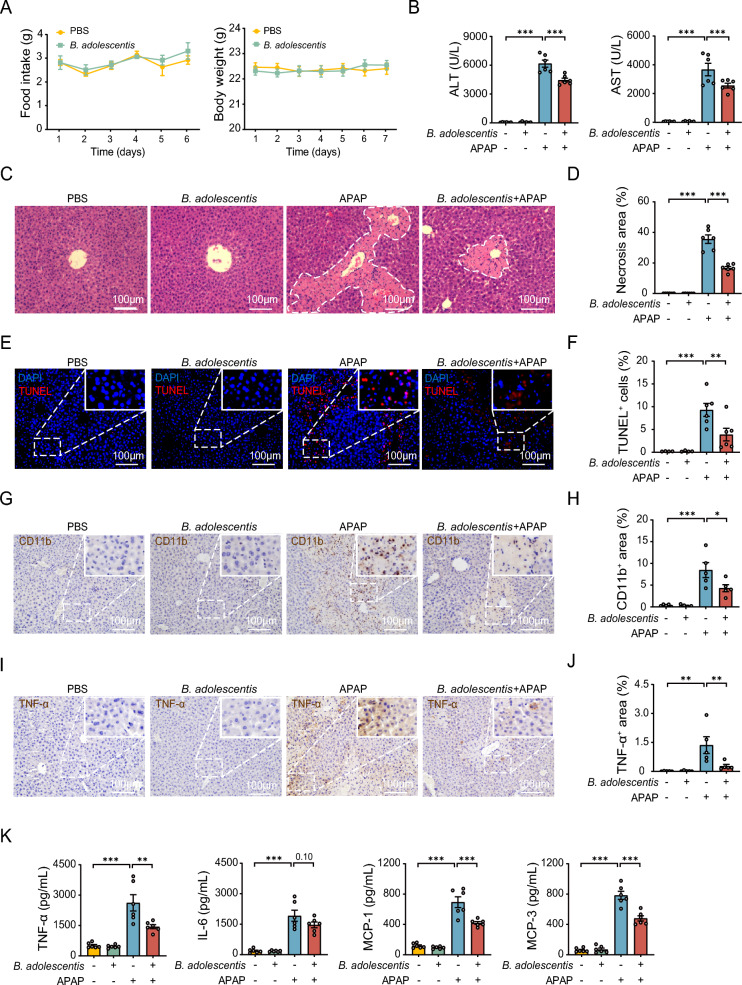
APAP-induced hepatotoxicity can be alleviated by *B. adolescentis* treatment in mice. **A** Body weight and food intake were measured daily in mice with or without *B. adolescentis* (2 * 10^8^ CFU per mouse) treatment for 7 days. *N* = 7–8. **B** Serum ALT and AST levels in APAP-treated mice with *B. adolescentis* pre-administration. Tissues were collected at 24 h after APAP exposure. *N* = 6. **C**, **D** H&E staining and quantification of necrotic areas in the liver of mice. The dashed lines indicate necrosis in centrilobular tissues. *N* = 6. **E**, **F** Intrahepatic cell death was detected by the TUNEL assay. TUNEL-positive cells are labeled in red. *N* = 4–6. **G**–**J** Immunohistochemical images and quantifications of TNF-α and CD11b positive areas in the liver. *N* = 5. **K** Serum pro-inflammatory cytokines and chemokines in APAP-treated mice with or without *B. adolescentis* treatment via enzyme-linked immunosorbent assay (ELISA). *N* = 6. All data are shown as the mean ± standard error of the mean. Comparisons were assessed by one-way ANOVA and Holm-Sidak post hoc tests. *p < 0.05, **p < 0.01, ***p < 0.001. Scale bars, 100 μm

To exclude the effect of *B. adolescentis* on energy metabolism, we first measured food intake and body weight over the experiment period and found no differences between the control and *B. adolescentis*-treated mice (Fig. 1A). As expected, the APAP group demonstrated markedly increased ALT and AST serum levels compared to the control mice, whereas pretreatment with *B. adolescentis* substantially reduced abnormal aminotransferase levels (Fig. 1B). Histopathological analysis showed that the control mice displayed no signs of liver damage, while the APAP group showed extensive inflammatory cell infiltration and severe liver necrosis. By contrast acute liver injury was reversed in the *B. adolescentis* group (Fig. 1C, D). Additionally, terminal deoxynucleotidyl transferase dUTP nick end labeling (TUNEL) staining also revealed that *B. adolescentis* decreased hepatocellular death in APAP-treated mice (Fig. 1E, F). In terms of inflammatory response, Immunohistochemistry (IHC) demonstrated a notable decrease in the positive area expressing tumor necrosis factor-α (TNF-α) and cluster of differentiation 11b (CD11b) within the livers of mice in the *B. adolescentis* group (Fig. 1G–J), suggestive of effective attenuation of inflammatory processes. The serum concentrations of inflammatory chemokines such as TNF-α, interleukin-6 (IL-6), monocyte chemoattractant protein-1 (MCP-1), and monocyte chemoattractant protein-1 (MCP-3), decreased with *B. adolescentis* treatment in the presence of APAP challenge (Fig. 1K). Therefore, administration of *B. adolescentis* significantly attenuates APAP-induced acute liver injury in mice.

### *B. adolescentis* directly generates hypaphorine

**Fig. 2:**
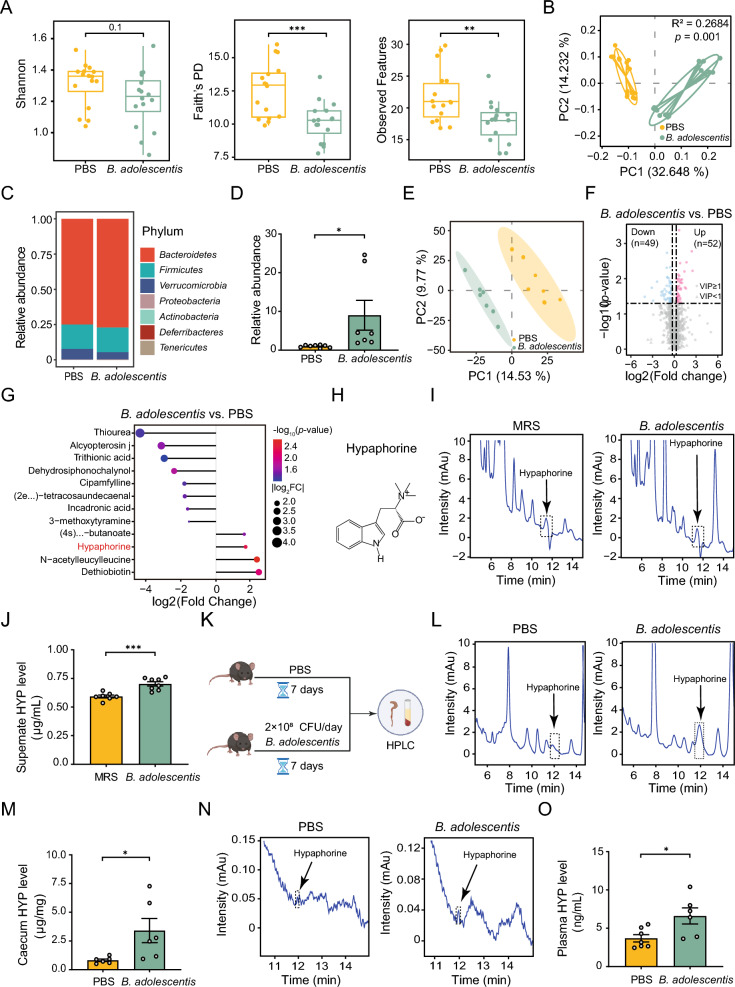
*B. adolescentis* can directly generate hypaphorine. **A** Alpha diversity of the gut microbiota in mice with or without *B. adolescentis* pre-treatment (2 * 10^8^ CFU per mouse) for 7 days. Data were measured using Shannon’s diversity index, Faith’s phylogenetic distance (PD), and observed features. *N* = 16. **B** The principal coordinates analysis (PCoA) of the gut microbiota in PBS or *B. adolescentis*-treated mice based on Bray–Curtis distance. *N* = 16. **C** Relative abundance in PBS or *B. adolescentis*-treated mice at the phylum level. *N* = 16. **D** The relative abundances of *B. adolescentis* in mice treated with PBS or *B. adolescentis* were quantified by qRT-PCR. *N* = 7–8. **E** Partial least squares discriminant analysis (PLS-DA) of metabolites in caecum samples from mice treated with PBS or *B. adolescentis*. *N* = 7–8. **F** Volcano plots depicting differential metabolites among PBS or *B. adolescentis*-treated mice. *N* = 7–8. **G** Lollipop plot of differential metabolites ranked by the fold change among PBS or *B. adolescentis*-treated mice. *N* = 7–8. **H** The chemical structure of hypaphorine. **I**, **J** Hypaphorine concentrations in culture supernatants of *B. adolescentis* or MRS were measured by HPLC. *N* = 7–8. **K**–**M** Hypaphorine concentrations of caecum in mice with or without *B. adolescentis* treatment were measured by HPLC. *N* = 6. **N**, **O** Hypaphorine concentrations of plasma in the mice with or without *B. adolescentis* treatment were measured by HPLC. *N* = 6–7. All data are shown as the mean ± standard error of the mean. Comparisons were assessed by two-tailed unpaired Student’s t-test. *p < 0.05, **p < 0.01, ***p < 0.001

To further investigate how *B. adolescentis* ameliorates APAP-induced liver injury, we first determined whether it colonized over 7 days. Following gavage with PBS or *B. adolescentis*, we collected mouse feces for 16S rRNA sequencing. According to the alpha diversity, the Faith's phylogenetic distance (PD) indexes and observed features changed discernibly, despite no significant difference in the Shannon index (Fig. 2A). Principal component analysis (PCoA) based on Bray Curtis distance matrices revealed a distinct separation in gut microbiota composition between control and *B. adolescentis*-treated mice (Fig. 2B). At the phylum level, there was a greater abundance of *Bacteroidetes* in *B. adolescentis*-treated mice compared with the control group (Fig. 2C). We also observed an increased abundance of *B. adolescentis* species by the quantitative real-time PCR (qRT-PCR) analysis (Fig. 2D). Next, we investigated which gut metabolite mediated the hepatoprotection of *B. adolescentis* against APAP-induced acute liver injury using untargeted metabolomics. Partial Least Squares Discrimination Analysis (PLS-DA) revealed significant differences in metabolite compositions between the *B. adolescentis* and PBS groups (Fig. 2E). Compared to the PBS group, 52 metabolites in *B. adolescentis* increased while 49 decreased (Fig. 2F). The top 3 upregulated metabolites were dethiobiotin, *N*-acetylleucylleucine, and hypaphorine (Fig. 2G). Hypaphorine is composed of three methyl groups and tryptophan (Fig. 2H). Numerous studies have shown that hypaphorine possesses antioxidant and anti-inflammatory properties [31, 32]. We therefore speculated that it might act as a potent protective metabolite of *B. adolescentis* against APAP-induced liver injury. To verify if *B. adolescentis can* directly generate hypaphorine, we collected the culture supernatants from *B. adolescentis* at 48 h and assessed them by high-performance liquid chromatography (HPLC) for hypaphorine production. As expected, the hypaphorine concentration was elevated in the *B. adolescentis* culture supernatants compared with the blank medium (Fig. 2I, J). Moreover, plasma and caecum hypaphorine levels were also elevated in mice treated with *B. adolescentis* (Fig. 2K–O). Based on the data above, we propose that the microbial metabolite hypaphorine is responsible for the hepatoprotection of *B. adolescentis*.

### Treatment with hypaphorine reduces APAP-induced acute liver injury in mice

**Fig. 3:**
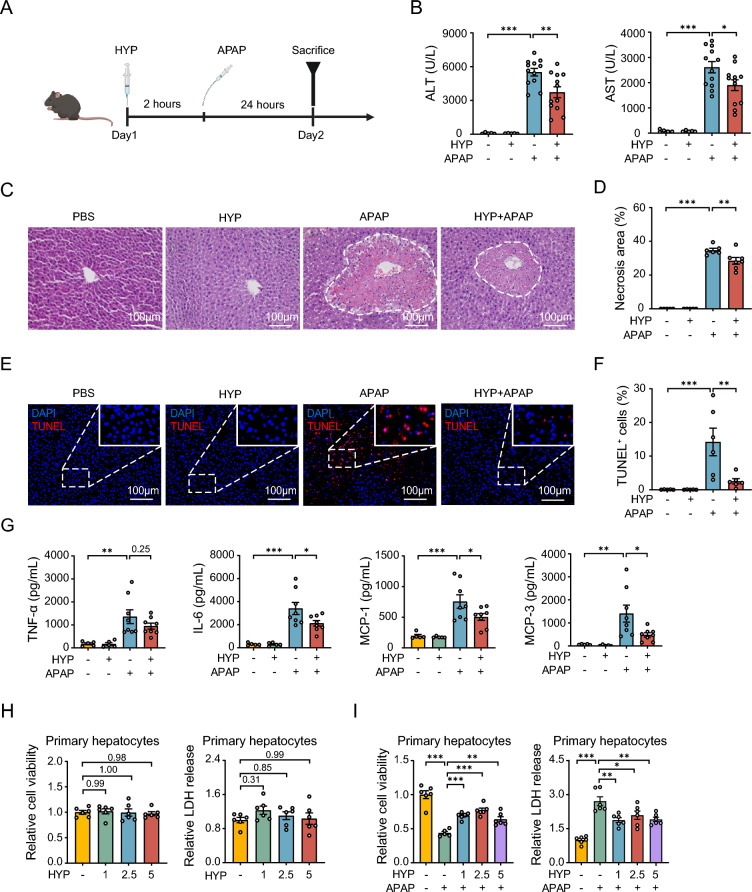
Treatment with hypaphorine reduces APAP-induced acute liver injury in mice. **A** A flowchart showing the experimental timeline of hypaphorine and APAP treatment. Mice were pretreated intraperitoneally with 10 mg/kg hypaphorine for 2 h, followed by subjection to oral administration of 300 mg/kg APAP. Tissues were collected at 24 h after APAP exposure. **B** Serum ALT and AST activities in the above mice. *N* = 5–12. **C**–**F** H&E and TUNEL staining were performed to quantify necrotic areas and cell death percentages in the liver. *N* = 5–7. **G** Quantitation of serum pro-inflammatory cytokines and chemokines by ELISA. *N* = 5–8. **H** CCK-8 and LDH release assays were performed to determine the viability of the primary hepatocytes treated with hypaphorine at concentrations of 1 μM, 2.5 μM, and 5 μM for 24 h. *N* = 6. **I** The relative viability of primary hepatocytes was detected by CCK-8 and LDH release assays. The primary hepatocytes were pretreated with hypaphorine for 2 h and then treated with 5 mM APAP for 24 h. *N* = 5–6. All data are shown as the mean ± standard error of the mean. Comparisons were assessed by one-way ANOVA with Holm-Sidak post hoc tests. *p < 0.05, **p < 0.01, ***p < 0.001. Scale bars, 100 μm

To verify our hypothesis, we pretreated mice with 10 mg/kg hypaphorine for 2 h and then treated with them APAP for 24 h. The ALT and AST serum levels were significantly decreased by hypaphorine pretreatment in APAP-treated mice (Fig. 3A, B). Haematoxylin and eosin (H&E) staining showed substantial reductions in necrotic lesions around the central liver veins in APAP-treated mice after hypaphorine pretreatment (Fig. 3C, D). TUNEL staining also demonstrated that hypaphorine decreased intrahepatic cell death in mice treated with APAP (Fig. 3E, F). Serum levels of TNF-α, IL-6, and MCP-1 were significantly decreased after hypaphorine pretreatment, while TNF-α decreased non-significantly (Fig. 3G). Additionally, we explored the protective effect of hypaphorine against AILI in primary hepatocytes by measuring CCK-8 and LDH release. As expected, hypaphorine at various concentrations had no toxicity but also significantly increased relative cell viability in APAP-treated primary hepatocytes (Fig. 3H, I). Together, the above data reveal that hypaphorine administration effectively mitigated liver injury caused by APAP.

### Hypaphorine restrains oxidative stress in the liver of APAP-treated mice

**Fig. 4:**
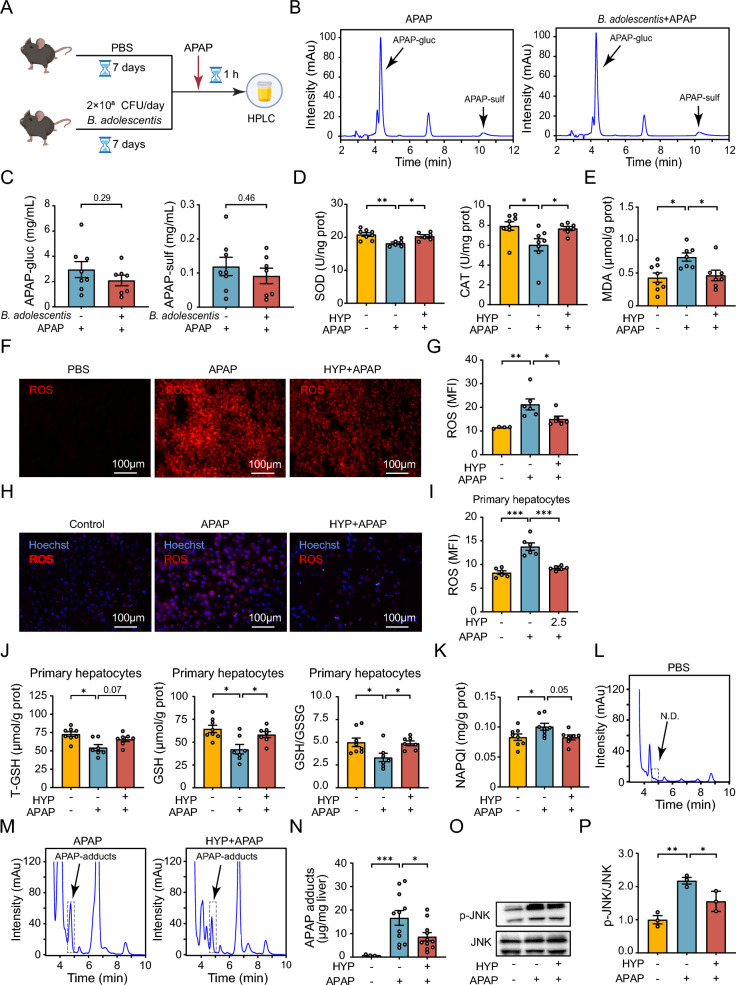
Hypaphorine restrains oxidative stress in the livers of APAP-treated mice. **A** Flow chart of the HPLC analysis. The mice were pretreated with or without *B. adolescentis* (2 × 10^8^ CFU per mouse) pretreated for 7 days, and then treated with APAP for 1 h. Urine samples were collected for HPLC analysis. **B**, **C** The APAP-glucuronide (APAP-gluc) metabolites and APAP-sulfate (APAP-sulf) metabolite levels. *N* = 7–8. **D**, **E** Hepatic levels of SOD, CAT, and MDA were detected in the mice pretreated with hypaphorine for 2 h, followed by treatment with APAP for 1 h. *N* = 6–8. **F**, **G** The levels of intracellular ROS in the livers were collected 1 h after APAP treatment. *N* = 4–6. **H**, **I** The ROS levels and mean fluorescence intensity of primary hepatocytes were measured after treatment with 2.5 μM hypaphorine for 2 h, followed by treatment with 5 mM APAP for 1 h. *N* = 6. **J** T-GSH and GSH levels and the GSH/GSSG ratio in primary hepatocytes with 2.5 μM hypaphorine pretreatment, followed by treatment with 5 mM APAP for 1 h. *N* = 7–8. **K** Hepatic NAPQI levels in the above mice. Livers were collected 1 h after APAP treatment. *N* = 7–8. **L**–**N** Quantification of hepatic APAP-adducts using HPLC in the above mice is performed 1 h after APAP treatment. *N* = 8–11. **O**, **P** The ratio of p-JNK/JNK in the liver of the mice was evaluated using Western blotting. Livers were collected 1 h after APAP treatment. *N* = 3. All data are shown as the mean ± standard error of the mean. Comparisons were assessed by two-tailed unpaired Student’s t-test (**C**) or one-way ANOVA with Holm-Sidak post hoc tests (**D**–**P**). *p < 0.05, **p < 0.01, ***p < 0.001. Scale bars, 100 μm

We next investigated the underlying mechanism of hypaphorine’s reduction in APAP-induced hepatotoxicity. We first conducted an HPLC analysis on urine samples collected from mice treated with APAP for 1 h with or without *B. adolescentis* pretreatment (Fig. 4A). We observed no differences in APAP-glucuronide (APAP-gluc) and APAP-sulfate (APAP-sulf) levels between the APAP-treated and APAP plus *B. adolescentis*-treated mice (Fig. 4B, C). Furthermore, the hepatic CYP2E1 and CYP1A2 protein levels remained stable with *B. adolescentis* pretreatment in the presence of an APAP challenge for 1 h (Figures S1A and S1B). These data suggest that *B. adolescentis*’ inhibition of AILI was independent of APAP metabolism. Moreover, hypaphorine did not affect liver cell nuclear proliferation, as indicated by the comparable levels of hepatic proliferating cell nuclear antigen (PCNA) between the two groups (Figure S1C). As oxidative stress plays a key role in AILI, we detected superoxide dismutase (SOD), catalase (CAT), and malondialdehyde (MDA) levels in the livers of mice exposed to APAP for 1 h with or without hypaphorine pretreatment. We observed significantly higher levels of SOD and CAT in the liver after hypaphorine treatment in the presence of an APAP challenge (Fig. 4D). Correspondingly, the hepatic MDA levels decreased by hypaphorine treatment in APAP-challenged mice (Fig. 4E). Hepatic reactive oxygen species (ROS) levels were decreased in hypaphorine plus APAP-treated mice (Fig. 4F, G). This was also demonstrated by the levels of ROS in the liver treated with *B. adolescentis.* We also observed beneficial effects in reducing hepatic ROS accumulation following the administration of *B. adolescentis* (Figure S1D). In vitro, ROS accumulation in APAP-treated primary hepatocytes was consistently reduced after hypaphorine pretreatment (Fig. 4H, I). We also assessed the glutathione (GSH) and GSH/GSSG ratio and found they markedly improved after hypaphorine treatment in APAP-stimulated primary hepatocytes (Fig. 4J). APAP is mainly oxidized by hepatic CYP450 to generate NAPQI and APAP-adducts, and it ultimately disrupts mitochondrial function and causes cell death [33]. We detected these intermediate metabolite levels and found that the mice pretreated with hypaphorine had lower levels of NAPQI and APAP-protein adducts in response to an APAP challenge (Fig. 4K–N). On the signaling molecules side, hypaphorine administration substantially decreased c-Jun N-terminal kinase (JNK) phosphorylation in the livers of the mice mentioned above (Fig. 4O, P). Hence, these results indicate that hypaphorine treatment suppresses intrahepatic oxidative stress in APAP-treated mice.

### The hepatic *Cry1* level was increased in APAP-treated mice after hypaphorine treatment

**Fig. 5:**
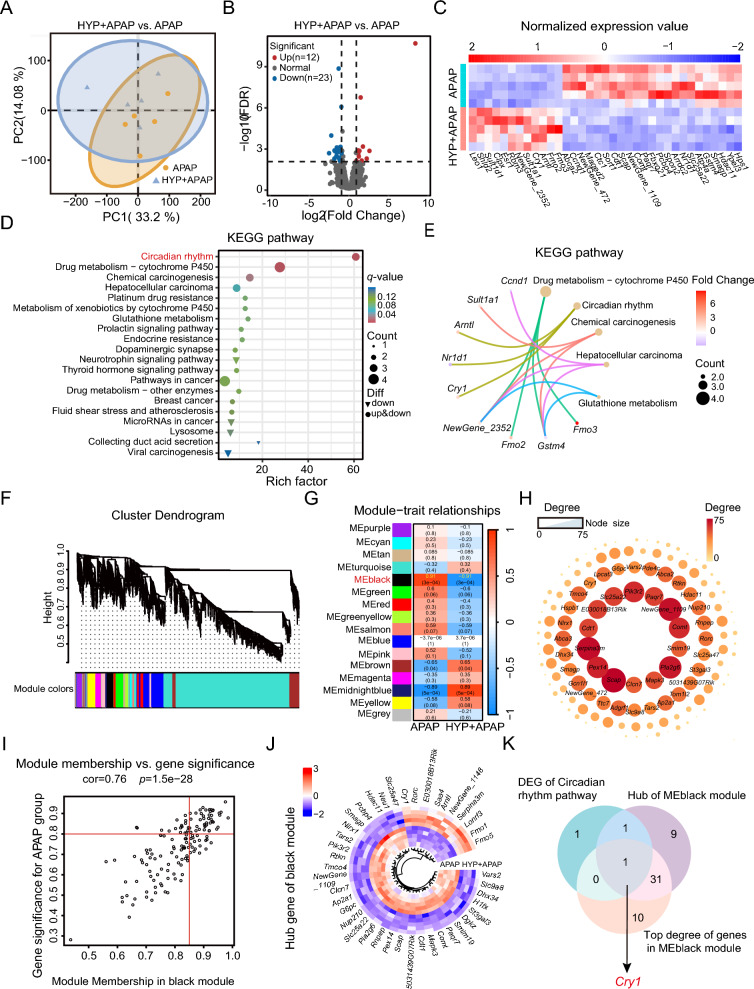
The hepatic *Cry1* level was increased in APAP-treated mice after hypaphorine treatment. **A** A PCA plot of transcriptomic analysis in primary hepatocytes isolated from APAP-treated mice with or without 10 mg/kg hypaphorine pre-administration. *N* = 5. **B** Volcano plots showed the 35 DEGs with fold changes greater than 2 and FDR < 0.01. *N* = 5. **C** Heatmap of differential gene cluster analysis shows the 35 DEGs in the primary hepatocytes. *N* = 5. **D** Pathway enrichment analysis of the KEGG pathways of the 35 DEGs in the primary hepatocytes. *N* = 5. **E** The top 5 pathways ranked by q-values in the KEGG pathway enrichment analysis. *N* = 5. **F** Modules based on the co-expression topological overlap within hierarchical clustering dendrograms from the top 5000 median absolute deviation genes by WGCNA. *N* = 5. **G** Pearson’s correlation coefficient was used to correlate module eigengenes with group traits. Each cell displays its correlation and *p*-value. *N* = 5. **H** The network plot of MEblack module membership. The inner circles are genes with a degree greater than 35. *N* = 5. **I** Scatterplots of gene significance for each MEblack module membership. *N* = 5. **J** Hub membership heatmap in the MEblack module. *N* = 5. **K** Venn diagrams showing an overlap of DEGs among the circadian rhythm pathway and hub or top 42 genes of the MEblack module. *N* = 5

We next investigated the specific gene involved in hypaphorine’s protection of APAP-induced liver injury by transcriptomics. We pretreated mice with or without hypaphorine for 2 h before administering APAP for 1 h and then we isolated primary hepatocytes for transcriptomic analysis (Figure S2A). The principal component analysis (PCA) plot shows differences in the global gene profiles between APAP-treated primary hepatocytes with or without hypaphorine treatment (Fig. 5A). We detected a total of 35 differentially expressed genes (DEGs) based on the criteria of |FC| ≥ 2 and FDR < 0.01 (Fig. 5B, C). Furthermore, gene ontology (GO) analysis revealed that the biological processes associated with the circadian regulation of gene expression were most significantly affected by hypaphorine treatment in the presence of an APAP challenge (Figures S2B, S2C). Meanwhile, KEGG analysis also revealed that the circadian rhythm pathway was the most significantly enriched among the two groups (Fig. 5D, E). To further identify potential core gene targets regulated by hypaphorine, we performed a weighted gene co-expression network analysis (WGCNA) analysis as described previously. We first transformed the gene expression data to screen the top 5000 genes in median absolute deviation and determined the soft threshold value of 7 for the best-fit polygenic (Figure S2D). Next, we used a dynamic tree-cut method and average hierarchical clustering to generate 16 modules. We focused on the MEblack module since it showed the highest correlation between the two groups (Fig. 5F, G). We then performed a gene network analysis of the 42 genes in the MEblack module with degrees greater than 35 using the R package and Cytoscape software (v3.10.1) (Fig. 5H). We screened for candidate hub genes by using the thresholds of the gene significance (|GS| > 0.8) and module membership (|MM| > 0.85), resulting in 42 genes screened in the upper-right corner (Fig. 5I, J). Finally, only the *Cry1* gene in the circadian rhythm pathway overlaps with the top degree and the hub genes in the MEblack module (Fig. 5K). This indicates that *Cry1* is a core gene involved in hypaphorine regulation of AILI.

### The *Cry1* gene activator KL001 alleviates APAP-induced acute liver injury

**Fig. 6:**
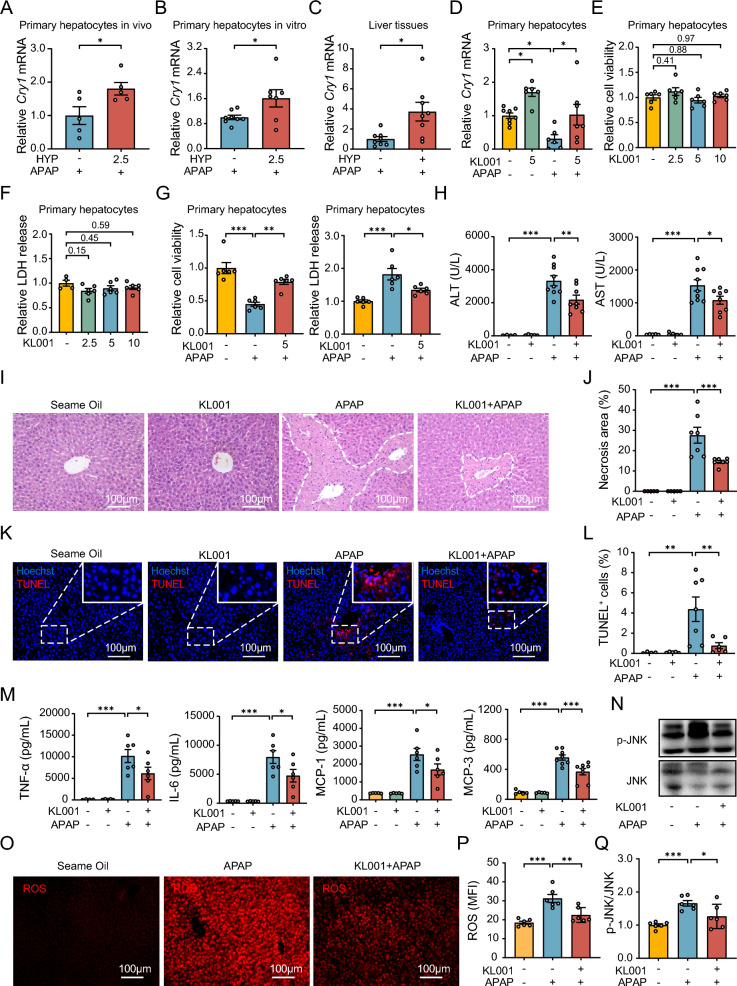
The *Cry1* gene activator KL001 alleviates APAP-induced acute liver injury. **A** The relative mRNA levels of *Cry1* in primary hepatocytes were determined by qRT-PCR. Primary hepatocytes were isolated from APAP-treated mice with or without 10 mg/kg of hypaphorine pretreatment. *N* = 5. **B** The relative *Cry1* mRNA levels were measured in primary hepatocytes treated with or without 2.5 μM hypaphorine for 2 h followed by 5 mM of APAP for 1 h. *N* = 7–8. **C** The relative *Cry1* mRNA levels in the liver were obtained from APAP-treated mice with or without 10 mg/kg hypaphorine pre-administration. *N* = 8. **D** The relative mRNA levels of *Cry1* were measured in primary hepatocytes treated with or without 5 μM KL001 for 2 h followed by 5 mM APAP for 1 h. *N* = 6–8. **E**, **F** The relative cell viability and LDH release were measured in primary hepatocytes with KL001 (2.5 μM, 5 μM, and 10 μM) treatment for 24 h. *N* = 4–6. **G** The relative cell viability and LDH release were measured in primary hepatocytes with or without KL001 and APAP treatment for 24 h. *N* = 6. **H** Serum ALT and AST levels in APAP-treated mice with 50 mg/kg KL001 intraperitoneal treatment for 2 h. Tissues were collected at 24 h after APAP exposure. *N* = 4–9. **I**, **J** H&E staining was performed to quantify necrotic areas in the livers of the mice. *N* = 5–7. **K**, **L** TUNEL staining was performed to quantify cell death percentages in the liver of the mice. *N* = 4–6. **M** Serum cytokine and chemokine concentrations of the mice. *N* = 5–8. **O**, **P** The hepatic levels of intracellular ROS were measured in mice after APAP for 1 h with KL001 pretreatment. *N* = 6. **N**, **Q** The p-JNK and JNK protein levels were assessed in livers of APAP-treated for 1 h mice with or without 50 mg/kg KL001 pretreatment. *N* = 6. All data are shown as the mean ± standard error of the mean. Comparisons were assessed by two-tailed unpaired Student’s t-test (**A**–**C)** or one-way ANOVA with Holm-Sidak post hoc tests (**D**–**Q**). *p < 0.05, **p < 0.01, ***p < 0.001. Scale bars, 100 μm

We next detected the changes in *Cry1* gene expression in response to hypaphorine treatment in the presence of an APAP challenge. First, we isolated the primary hepatocytes of APAP-treated mice with or without hypaphorine pretreatment and found that *Cry1* gene expression was significantly increased by hypaphorine treatment (Fig. 6A). A similar result was seen in primary hepatocytes isolated from normal mice and then treated with hypaphorine plus APAP (Fig. 6B). We also found that hypaphorine treatment significantly upregulated *Cry1* gene expression in the presence of an APAP challenge in mice (Fig. 6C). Therefore, we performed further experiments using the KL001 stabilizer, which greatly activated *Cry1* gene expression (Fig. 6D). The cell viability and LDH release of primary hepatocytes was not affected when KL001 was administered at concentrations of 2.5 μM, 5 μM, and 10 μM (Fig. 6E, F). However, KL001 dramatically ameliorated APAP-induced decrease in cell viability (Fig. 6G). We also investigated whether *Cry1* gene activation exerted the same hepatoprotection in APAP-treated mice. The biochemical results showed that KL001 treatment significantly reduced ALT and AST serum levels in mice facing an APAP challenge (Fig. 6H). H&E and TUNEL staining indicated that KL001 effectively reduced the area of liver necrosis and cell death (Fig. 6I–L). Moreover, the lower serum concentration of inflammatory chemokines, such as TNF-α, IL-6, MCP-1, and MCP-3, of mice treated with APAP plus and KL001 further supports *Cry1*’s protective effects (Fig. 6M). CYP2E1, CYP1A2, and PCNA protein levels stabilized in KL001 plus APAP-treated mice compared to the APAP group (Figures S3A–S3C). KL001 treatment both reduced hepatic ROS and p-JNK levels in APAP-treated mice (Fig. 6N–Q), suggesting that KL001 improves the hepatocyte’s antioxidant capacity to resist APAP-induced liver injury. This is further supported by ROS and GSH levels in the primary hepatocytes (Figures S3D–S3F). In brief, all the above data showed that the *Cry1* gene activator KL001 protects against APAP-induced acute liver injury by reducing oxidative stress.

### Decreased *CRY1* gene levels in APAP-induced patients with ALF

**Fig. 7:**
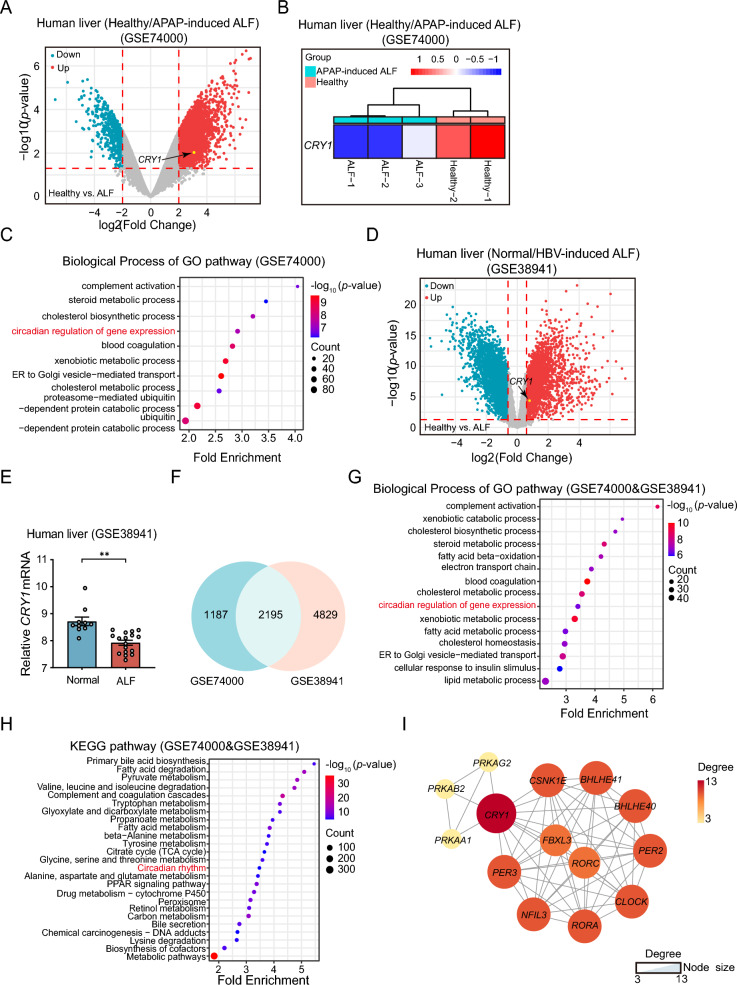
Decreased *CRY1* gene levels in APAP-induced patients with ALF. **A** Volcano plots of DGEs with |log_2_Fold Change| > 2 and *p* < 0.05 in the livers of healthy humans and patients with AILI from the GSE74000 data set. *N* = 2–3. **B** The heatmap of *Cry1* genes expressed in the livers of healthy humans and those with AILI from the GSE74000 data set. *N* = 2–3. **C** The GO pathway enrichment of hepatic DEGs of healthy human subjects and those with AILI from the GSE74000 data set. *N* = 2–3. **D** Volcano plots of DGEs with |log_2_Fold Change| > 0.6 and *p* < 0.05 in the livers of healthy humans and patients with HBV-induced ALF from the GSE38941 data set. *N* = 10–17. **E** Hepatic *Cry1* mRNA expression levels in healthy humans and those with HBV-induced ALF from the GSE38941 data set. *N* = 10–17. **F** Venn diagrams show the overlapping DEGs in the GSE74000 and GSE38941 datasets. **G**, **H** The GO and KEGG pathways enrichment plot of the overlapping DEGs obtained from the Venn diagram analysis. **I** The network plot of 14 genes in the circadian rhythm pathway. Nodes are arranged in a circular mode by degree value (size and color depth remain proportional to the degree). All data are shown as the mean ± standard error of the mean. Comparisons were assessed by two-tailed unpaired Student’s t-test (**E**). *p < 0.05, **p < 0.01, ***p < 0.001. Scale bars, 100 μm

To excavate *CRY1* gene expression in APAP-induced patients with ALF, we analyzed the GSE74000 and GSE38941 data sets from the NCBI’s GEO database (https://www.ncbi.nlm.nih.gov/) using R studio software. As shown in Fig. 7A, B, the mRNA levels of *Cry1* in the healthy population were significantly higher than those in the APAP-induced ALF group. We conducted a GO enrichment analysis of the DEG processes using the DAVID database (https://david.ncifcrf.gov/). We found that the biological processes associated with circadian regulation of gene expression ranked fourth in fold enrichment (Fig. 7C). We also analyzed the GSE38941 data set regarding HBV-induced ALF. Compared with the HBV-induced ALF group, *CRY1* genes were significantly upregulated in the healthy populations (Fig. 7D, E). As we examined 2195 DEGs that intersected in the two data sets, we found that they were significantly enriched in the circadian regulation of gene expression among the two groups (Fig. 7F, G). PPI networks constructed with the 14 DEGs reveal that the *CRY1* gene occupies the core of the circadian rhythm pathways (Fig. 7H, I). In conclusion, the above results indicated that *CRY1* gene expression was significantly reduced following ALF, regardless of whether APAP or HBV were induced.

## Discussion

Despite its popularity as an antipyretic and analgesic, excessive use of APAP is increasingly recognized as a potential causative liver damage agent [34, 35]. Conventional AILI treatment involves oral or intravenous NAC, but its short application window and potential to cause adverse gastrointestinal events present challenges [36]. Research on the gut-liver axis has recently garnered significant attention. Liver disease is often concurrent with a disturbance in the gut microbiota [37–39]. As previously demonstrated, cisplatin disrupts the intestinal microbiota, leading to increased inflammation and oxidative stress, which exacerbate hepatotoxicity [40]. Probiotics protect the liver by maintaining gut barrier integrity and promoting host metabolism [41, 42]. Our recent study revealed that mice receiving *Lactobacillus vaginalis* were not susceptible to APAP hepatotoxicity [11]. In addition, *B. adolescentis* has a protective effect in various liver diseases. It may protect against nonalcoholic fatty liver disease by enhancing fibroblast growth factor 21 (FGF21) sensitivity [43]. In this study, we demonstrate that treatment with *B. adolescentis* significantly reduces the susceptibility of mice to AILI, as indicated by the decreased ALT and AST serum levels. Furthermore, the histopathological and inflammatory cytokines results proved that *B. adolescentis* effectively protects against AILI by reducing hepatocellular death and inflammatory factors in APAP-treated mice. Our observation provides a novel perspective for overcoming AILI. However, further research is warranted to determine its clinical effectiveness.

In this study, we successfully observed the colonization of *B. adolescentis* in mice after gavaging them with *B. adolescentis* (2 × 10^8^ CFU per mouse) for 7 days. Diversity results revealed a notable reduction in gut microbiota abundance following the intervention. Walker et al. reported that the composition of the gut microbiota can be influenced by dominant species, which can lead to potential health consequences [44]. For example, *Bifidobacterium* effectively prevents pathogen colonization and vigorously fights pathogen infection [45, 46]. *Bifidobacteria* competitively exclude *Campylobacter jejuni* colonization in mice and counteract *Salmonella*-induced immune suppression in mice [47]. Colonization of *B. adolescentis* in mice may lead to a significant reduction in the diversity or abundance of the gut microbiota. This phenomenon may be attributed to the complexity and dynamics of the gut microbiome, as well as the possible existence of competitive exclusion mechanisms. However, these conjectures and hypotheses need to be further verified and confirmed in subsequent experiments. The ratio of Firmicutes to Bacteroidetes (F/B) is crucial for maintaining homeostasis, and its variation can influence the overall stability and function of the intestinal microbiota [48]. In our study, despite noting an upward trend in the abundance of Bacteroidetes, qRT-PCR analysis demonstrated no significant alteration in the F/B ratio. This suggests that the administration of *B. adolescentis* did not markedly impact the F/B ratio within the mouse gut microbiota, thereby preserving the homeostasis and health of the intestinal tract in mice to a certain degree. However, to further explore the influence of *B. adolescentis* on mouse gut microbiota structure and function, germ-free mice experiments are typically necessary, and we will incorporate this experiment in our future work.

Next, considering AILI therapy’s possible reliance on the release of gut-derived metabolites, we conducted a metabolomics analysis and found that *B. adolescentis* induces hypaphorine production. Hypaphorine can be extracted from the microbiome and also *Abrus cantoniensis Hance* (ACH), *Callerya speciosa*, and *Abrus precatorius* L. [22, 49, 50]. Furthermore, the consumption of specific foods, particularly chickpeas and lentils, induces the production of hypaphorine. It can be detected as a biomarker of food intake (BFI) in the serum and urine samples of volunteers [51]. Lactating mothers who consume legumes rich in hypaphorine, such as peanuts, can also have hypaphorine detected in their breast milk [52]. Additionally, hypaphorine is one of the numerous microbial metabolites associated with food that have been discovered in plant-based diets [53]. Prior studies have shown that *Lactobacillus vaginalis* releases daidzein from dietary components via β-galactosidase [11]. Based on our study, which has confirmed that the ingestion of *B. adolescentis* can enhance the production of hypaphorine, we speculate that hypaphorine may undergo metabolism by *B. adolescentis*, facilitated by specific enzymes. However, this speculation requires further investigation for validation.

Apart from its diverse sources, hypaphorine is highly bioavailable and shows great potential in medicine [54]. For example, ACH decreases inflammation, improves antioxidant capacities, and regulates intestinal bacterial populations, thereby alleviating CCl_4_-induced liver injury [55]. A recent study has revealed an inverse correlation between metabolites associated with plant-based diet indices and metabolic syndrome. This suggests that metabolites such as hypaphorine may be crucial targets in plant-based diets’ ability to mitigate cardiometabolic risks [53]. Herein, we provide good evidence for the effectiveness of hypaphorine in APAP treatment hepatotoxicity. According to our in vitro and in vivo experiments, hypaphorine pretreatment results in greater resistance to acute liver injury. Furthermore, as mitochondrial oxidative stress is a hallmark of AILI [56], we observed significant reductions in oxidative stress indicators such as MDA and SOD in APAP-treated mice after hypaphorine treatment. This resulted in AILI alleviation, which was further corroborated by the reduction of ROS accumulation, APAP-adducts, and p-JNK proteins in the liver. Taken together, our research unveils the promising therapeutic potential of hypaphorine as a groundbreaking treatment strategy for AILI. Further exploration of its clinical efficacy is warranted.

Finally, our findings revealed a significant role of the *Cry1* gene in APAP-induced hepatic oxidative stress in mice. It is well known that *Cry1* is involving in regulating the circadian period. Circadian rhythms are closely associated with the development of APAP hepatotoxicity [57]. This was demonstrated by our previous study, which showed that acute liver damage was more severe in mice administered APAP at night compared to during the day [29]. The current mechanism suggests that the hepatocyte circadian clock regulates APAP hepatotoxicity by controlling cytochrome P450-dependent activity through NADPH-cytochrome P450 oxidoreductase [58]. Based on our transcriptomics data, we found that only the *Cry1* gene in the circadian rhythm pathway overlaps with the top degree and hub genes in the MEblack module. Notably, qRT-PCR analysis revealed a marked elevation in *Cry1* gene expression with hypaphorine treatment in mice, reinforcing the validity of our findings. In addition, we confirmed that KL001, a *Cry1* stabilizer, can mitigate oxidative stress and reduce hepatocyte injury in mice. We also analyzed the GSE7400 and GSE38941 datasets, demonstrating that patients with liver disease have low expression of the hepatic *Cry1* gene.

In conclusion, our research reveals that *B. adolescentis* protects against AILI by producing the hypaphorine metabolite, which regulates *Cry1* gene expression. This protective effect could be attributed to a reduction in inflammatory and oxidative stress. These findings offer novel insights into the potential therapeutic application of *B. adolescentis* for AILI management.

## Materials and methods

### Animal models

Eight-week-old C57BL/6J mice were obtained from Liaoning Changsheng Biotechnology Co., Ltd (China). Mice were placed in standard lab conditions with access to water, food, and 12-h light/dark cycles. Acute liver injury was induced by oral administration of 300 mg/kg APAP (Macklin, Shanghai, China) at 8:00 PM for 24 h. For the *B. adolescentis* intervention, we mice were gavaged with *B. adolescentis* (2 × 10^8^ CFU per mouse) for 7 consecutive days, followed by APAP treatment. For small molecule treatment, hypaphorine (10 mg/kg, Macklin) or KL001 (50 mg/kg, Macklin) were injected intraperitoneally for 2 h before the APAP challenge. All experimental procedures were approved by the local animal care and use committee of Guangdong Pharmaceutical University.

### Bacterial culture

*B. adolescentis* was grown anaerobically in de Man, Rogosa, and Sharpe (MRS, Hopebio, Qingdao, China) broth and passaged every 2 days. Species identification was conducted using the PubMed Nucleotide BLAST database (https://blast.ncbi.nlm.nih.gov).

### Primary hepatocyte isolation and culture

The mouse primary hepatocytes were isolated using collagenase digestion as previously described [59]. Briefly, a warm buffer solution was used to eliminate blood cells from mice livers, followed by hepatocyte dissociation with type IV collagenase (Worthington, NJ, USA). Hepatocytes from dissected livers and inoculated them into collagen-coated dishes (Corning, NY, USA). Afterward, 10% fetal bovine serum (Gibco, CA, USA) was added along with 100 U/mL penicillin/streptomycin in RPMI1640 culture medium (Gibco, CA, USA) for 6 h in the incubator (Heal Force HF100, Hong Kong, China). For hypaphorine or KL001 intervention, primary hepatocytes were treated with various concentrations of hypaphorine (1 μM, 2.5 μM, and 5 μM) or KL001 (2.5 μM, 5 μM, and 10 μM) for 24 h. For the APAP treatment, primary hepatocytes were treated with 5 mM APAP for 24 h.

### Biochemical and ELISA assays

The serum levels of ALT and AST were measured with a commercial kit (Nanjing Jiancheng Bioengineering Institute, Nanjing, China). As instructed by the manufacturer, commercial assay kits were used to measure SOD (Nanjing Jiancheng Bioengineering Institute, Nanjing, China), CAT (Nanjing Jiancheng Bioengineering Institute, Nanjing, China), MDA (Beyotime, Shanghai, China), and NAPQI (JSBOSSEN, Jiangsu, China) activities in homogenate liver tissues, as well as the levels of GSSG (Nanjing Jiancheng Bioengineering Institute, Nanjing, China) and T-GSH (Nanjing Jiancheng Bioengineering Institute, Nanjing, China) levels in the primary hepatocytes. Cell viability and LDH release in primary hepatocytes were assessed by the Cell Counting Kit-8 (Meilunbio, Dalian, China) and CytoTox 96® Non-Radioactive Cytotoxicity Assay Kits (Promega, WI, USA), respectively. The serum levels of TNF-α (Neobioscience, Shenzhen, China), IL-6 (Neobioscience, Shenzhen, China), MCP-1 (Neobioscience, Shenzhen, China), and MCP-3 (CUSABIO, Wuhan, China) were determined with ELISA kits.

### Histopathological examination, reactive oxygen species level determination and immunohistochemistry

For the H&E staining, mouse livers were fixed in 4% paraformaldehyde fixative for 24 h, followed by dehydrated, clarified, wax embedded, sectioned, and baked at 60 °C for 1 h. To assess cell death, a commercial kit (KeyGEN BioTECH, Nanjing, China) was used for TUNEL staining. Detection of reactive oxygen species (ROS) in the primary hepatocytes and liver tissues was detected with the Hoechst assay kit (Beyotime, Shanghai, China) and Dihydroethidium (DHE, Thermo Science, MA, USA), respectively. For immunohistochemistry experiments, a specially formulated citrate buffer, containing 10 mM sodium citrate and 0.5% Tween 20 at a pH of 6.0, is used to facilitate endogenous repair. Each slide was incubated with specific TNF-α (Affinity, Jiangsu, China) or Anti-CD11b Rabbit pAb (Servicebio, Wuhan, China) antibodies at 4 °C overnight, and then incubated with a secondary antibody (Servicebio, Wuhan, China) at room temperature for 1 h. Diaminobenzidine tetrahydrochloride (DAB, ZSGB-BIO, Beijing, China) was used as a chromogenic agent to visualize the antigen–antibody complexes. Nuclei were then counterstained with hematoxylin, and tissue sections were dehydrated for structural stability. Finally, the slides were mounted for subsequent microscopic evaluation and analysis. Each of the 5–8 random visual field slices was captured under a microscope (Leica DMi8, Wetzlar), and analyzed using Image J software (National Institutes of Health).

### Quantitative reverse transcription PCR (qRT-PCR)

The total RNA was extracted using the TRIzol reagent (Invitrogen, CA, USA) and reverse transcribed using a reverse transcription reagent kit (Toyobo, Osaka, Japan). The amplification of cDNA was conducted using specific primers and the SYBR Green PCR Master Mix (Toyobo, Osaka, Japan) on the Light Cycler 96 (Roche). With the comparative Ct method, relative mRNA expression was quantified by normalizing each sample for RNA amounts with *18S*. Table 1 provides an overview of the qRT-PCR primers.

### Protein extraction and Western blotting

Total proteins were prepared using the RIPA lysis buffer (Beyotime, Shanghai, China) that contained phosphatase and protease inhibitors. SDS-PAGE was used to separate protein samples, followed by transfer onto nitrocellulose membranes (Merck, MA, USA). After blocking the bands for 1 h in 5% non-fat dry milk (Solarbio, Beijing, China) at room temperature, the bands were incubated with different primary antibodies, such as the SAPK/JNK (Cell Signaling Technology, MA, USA), phospho-SAPK/JNK (Thr183/Tyr185) (Cell Signaling Technology, MA, USA), CYP1A2-specific polyclonal (Affinity, Jiangsu, China), CYP2E1-specific polyclonal (Proteintech, Wuhan, China), PCNA (Abmart, Shanghai, China), β-actin (Servicebio, Wuhan, China), GAPDH (Servicebio, Wuhan, China) antibodies, at 4 °C overnight. Following that, the protein bands were incubated with a secondary antibody (Cell Signaling Technology, MA, USA) at room temperature for 1 h, and the protein bands were detected by an enhanced chemiluminescence assay (ECL, Biosharp, Beijing, China). The relative protein expression was statistically analyzed using the Image J software. Table 2 provides an overview of the key resources.

### 16S rRNA gene sequencing analysis

Feces DNA was extracted with a DNA isolation kit (MaBio, Guangzhou, China), measured with Epoch microplate readers (BioTek, Winooski, VT, USA) for purity and concentration, and then diluted to 10 ng/L. The reaction system consisted of a 12 μL reaction tube containing template DNA (5 μL), 1 μL 16S amplicon primers (forward primer: 5′-GTGTGYCAGCMGCCGCGGTAA-3′; reverse primer: 5′-CCGGACTACNVGGGTWTCTAAT-3′), and 6 μL SYBR Green Mix. The qRT-PCR program was executed on a 7500 Real-Time PCR System (Applied Biosystems, MA, USA). The PCR products were sequenced on Illumina NovaSeq 6000 platforms. Data were analyzed in bioinformatics by QIIME 2.

### Metabolomics sequencing

For metabolomic analysis, cecal contents were obtained from control or *B. adolescentis-*treated mice. The Metabolomics sequencing was commercially processed by Beijing Genomics Institution (China). Raw data was imported into Compound Discoverer 3.3 (Thermo Fisher Scientific, USA) and combined with the BGI Metabolome Database, mzCloud database, and ChemSpider online database for analysis.

### HPLC analysis

For hypaphorine detection, *B. adolescentis* was cultured in MRS for 48 h at 37 °C and then the sample was centrifuged at 8000 rpm for 5 min to obtain bacterial supernatant. All samples were extracted with methanol in a weight/volume ratio of 1:10 and centrifuged at 13,000 rpm for 10 min at 4 °C. The mobile phase was acetonitrile and 0.1% (volume/volume) formic acid water at a volume ratio of 10:90 and a flow rate of 1.0 mL/min for 15 min. The measurement was performed on 1260 HPLC (Agilent, CA, USA). To detect APAP-protein adduct levels, the sample preparation follows the above procedure, but the mobile phase was adjusted to methanol and 0.1% (volume/volume) formic acid water at a volume ratio of 20:80 for 10 min. To detect APAP-glucuronide (APAP-gluc) metabolites and APAP-sulfate (APAP-sulf) metabolite levels, urine samples were processed with methanol and a mixture of 1:400 (weight/volume) and then centrifuged at 13,000 rpm for 10 min. The mobile phase was acetonitrile and 0.1% (volume/volume) formic acid water at a volume of 10:90 and a flow rate of 1.0 mL/min for 15 min. All samples were dried in a vacuum concentrator (Eppendorf Concentrator Plus, Eppendorf, Hamburg, Germany) and redissolved in methanol. A volume of 10 μL sample was injected into the Cosmosil-C18 column (250 mm × 4.6 mm, 5 μm, Nacalai Tesque, Kyoto, Japan). Acquired and processed data were handled by Agilent LC1260 software.

### Transcriptomic sequencing

For transcriptomic analysis, mice were injected intraperitoneally 10 mg/kg hypaphorine 2 h before administering 300 mg/kg of APAP. The primary hepatocyte was isolated after 1 h of APAP treatment and the RNA was extracted using a TRIzol reagent (Invitrogen, CA, USA). Following quality control of total RNA, the cDNA library for sequencing by Biomarker Technologies Co., Ltd. (Beijing, China) using an Illumina HiSeq platform. DEGs were determined by Fold Change ≥ 2 with a false discovery rate (FDR) < 0.01. The DEGs were analyzed using the GO and KEGG pathways. To further identify the core target genes, the top 5000 genes were selected based on their median absolute deviation and processed with WGCNA of R-studio (v.4.3.1), as previously described [60]. Finally, we analyzed the gene network diagram in the screened modules using the STRING database (https://string-db.org) and Cytoscape software (v3.10.1).

### Data processing

Publicly available data sets (GSE74000 and GSE38941) were obtained from the GEO database (https://www.ncbi.nlm.nih.gov/geo/) and analyzed using R-studio (v.4.3.1). DEGs are defined as |log_2_Fold Change| > 2 with *p* < 0.05 in GSE74000 and |log_2_Fold Change| > 0.6 with *p* < 0.05 in GSE38941. GO enrichment and KEGG analyses are conducted using the DAVID database (https://david.ncifcrf.gov/). DEG intersections between the above data sets were further analyzed. Afterward, the data was visualized on the bioinformatics website (http://www.bioinformatics.com.cn/) and Cytoscape software (v3.10.1).

### Statistical analysis

Statistical analyses were performed using GraphPad Prism 8 software (Graph-Pad, San Diego, CA). All data are shown as the mean ± standard error of the mean. Comparisons were assessed by two-tailed unpaired Student’s t-test or one-way ANOVA with Holm-Sidak post hoc tests. Other statistical methods were given in the Figure legends. *p < 0.05, **p < 0.01, ***p < 0.001. BioRender (http://biorender.com/) was used to create the flow charts.

### Supplementary Information

Supplementary Material 1.

## Data Availability

The 16S sequence, metabolome and transcriptome data were deposited in the CNGBdb: CNP0005177, CNP0005160 and CNP0005162. The data are included in the article as figures, tables, and others which can be obtained from the corresponding author via email upon reasonable request.
