# Supplementary material for: Bifidobacterium adolescentis-derived hypaphorine alleviates acetaminophen hepatotoxicity by promoting hepatic Cry1 expression
Source: J Transl Med. 2024 May 31;22:525. doi: 10.1186/s12967-024-05312-6 (PMC11143572; doi:10.1186/s12967-024-05312-6)
Supplement: Supplementary file 1 — Supplementary Material 1. [file 12967_2024_5312_MOESM1_ESM.pdf]

Figure S1

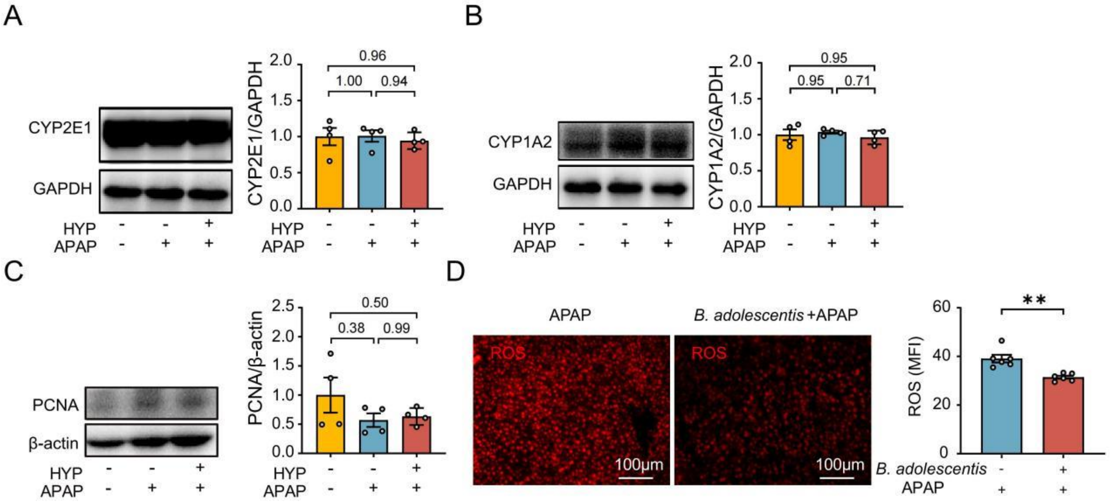

Figure S1. ***B. adolescentis*-derived hypaphorine restrains oxidative stress in the livers of APAP-treated mice**

(A and B) Western blotting was performed to detect CYP2E1 and CYP1A2 in the livers of APAP-treated mice with or without 10 mg/kg hypaphorine for 2 hours. Liver tissues were collected 1 hour after APAP treatment. *N* = 4. (C) Western blotting was performed to detect PCNA in the livers of APAP-treated mice with or without 10 mg/kg hypaphorine for 2 hours. Liver tissues were collected 24 hours after APAP treatment. *N* = 4. (D) The ROS levels in liver tissues after 1 hour of APAP treatment with *B. adolescentis* pretreatment. Liver tissues were collected 1 hour after APAP treatment. *N* = 6. All data are shown as the mean ± standard error of the mean. Comparisons were assessed by one-way ANOVA with Holm-Sidak post hoc tests (A-C) or two-tailed unpaired Student's t-test (D). *$p < 0.05$, **$p < 0.01$, ***$p < 0.001$.

Figure S2

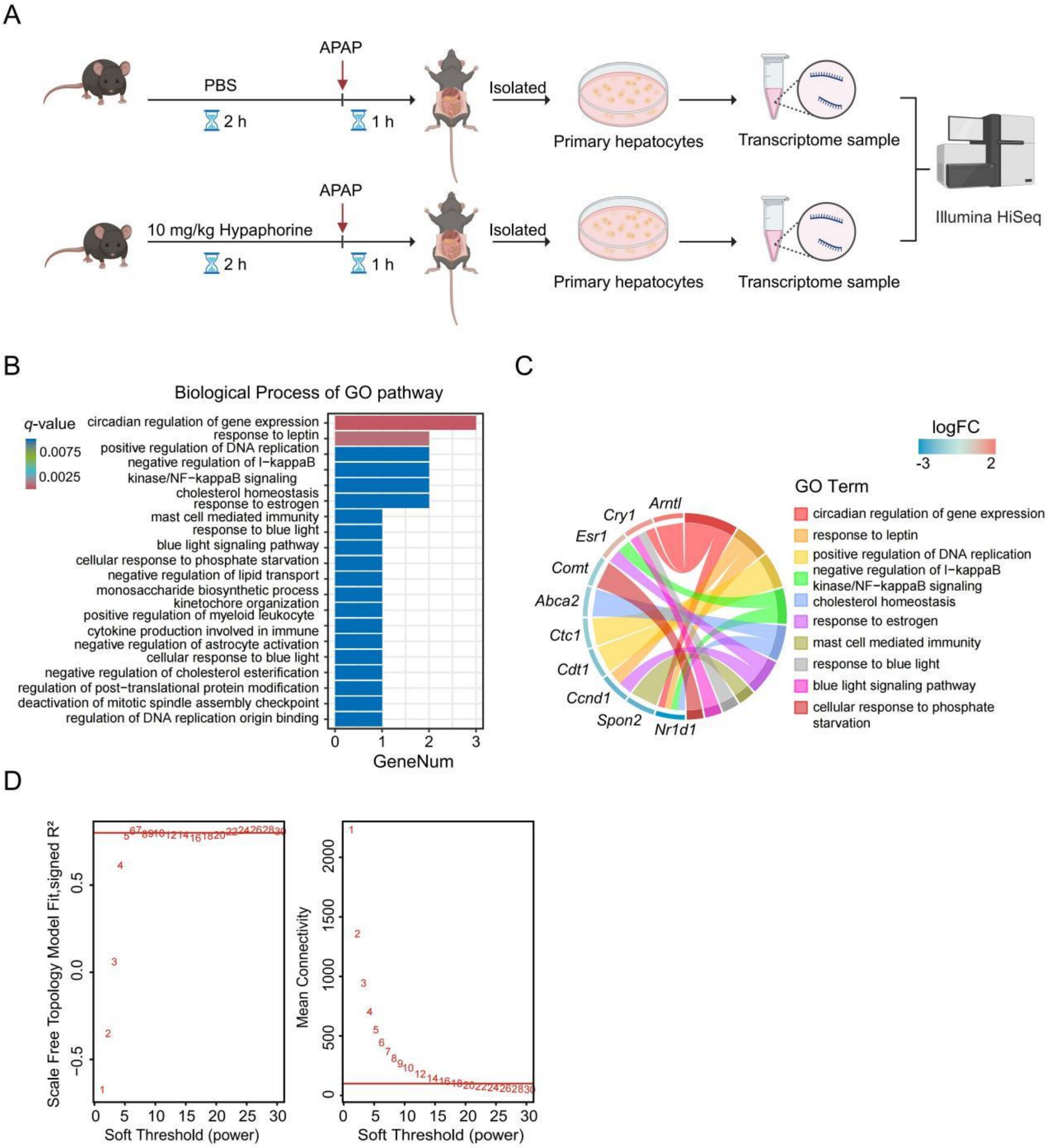

Figure S2. The hepatic *Cry1* level was increased in APAP-treated mice after hypaphorine treatment

(A) A flow chart of transcriptome sequencing sample preparation in primary hepatocytes. Mice were treated with PBS or 10 mg/kg hypaphorine for 2 h, and then treated with APAP for 1 h. Primary hepatocytes were isolated for RNA sequencing. (B) The enriched GO biological processes pathways of the 35 DEGs in the primary are described above. *N* = 5. (C) The chord diagram of the top 10 enriched GO terms of the 35 DEGs. *N* = 5. (D) Fit index and mean

connectivity for a scale-free topology model under different soft thresholds. The soft-threshold power was set at 7 and the cutoff at 0.8 (red line). *N* = 5.

Figure S3

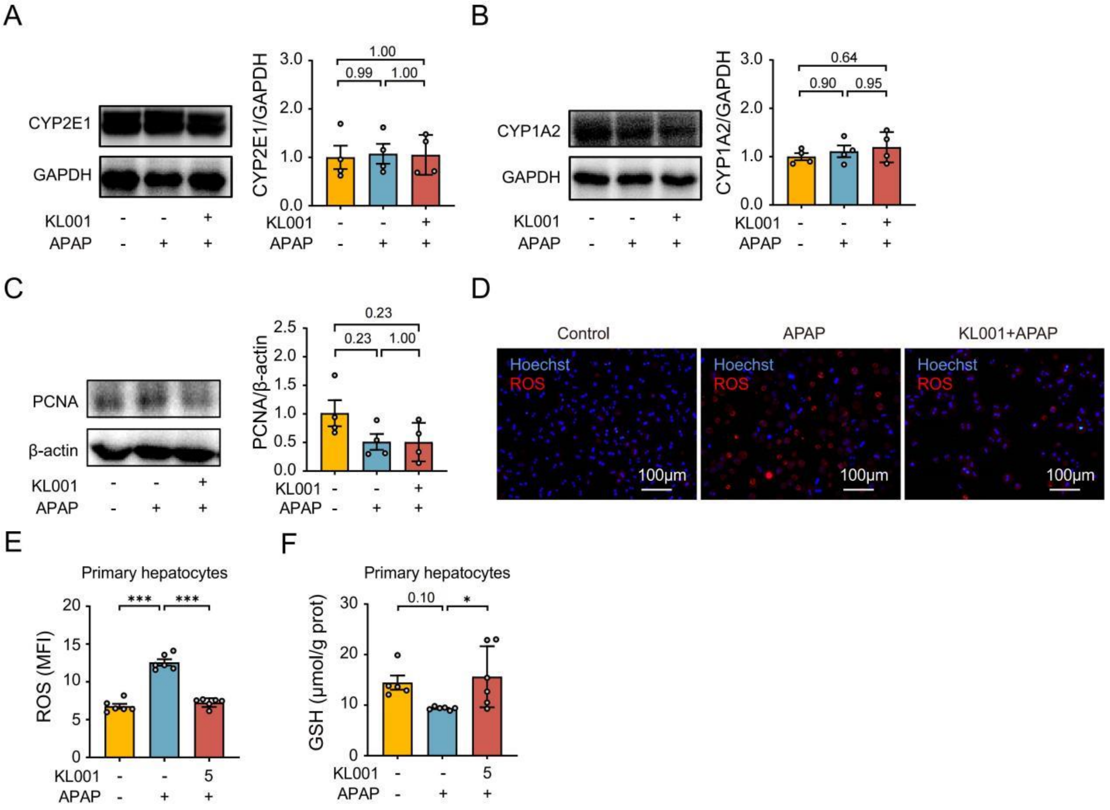

Figure S3. The *Cry1* gene activator KL001 alleviates APAP-induced acute liver injury

(A and B) Western blotting was used to detect CYP2E1 and CYP1A2 protein expression in livers of APAP-treated mice with or without 50 mg/kg KL001 pretreatment for 2 h. Liver tissues were collected at 1 h after APAP treatment. *N* = 4. (C) Western blotting was used to detect PCNA protein expression in livers of APAP-treated mice with or without 50 mg/kg KL001 pretreatment for 2 h. Liver tissues were collected at 24 h after APAP treatment. *N* = 4. (D and E) Intracellular ROS levels in primary hepatocytes after APAP 1 with 5 μM KL001 pretreatment. *N* = 6. (F) GSH levels in primary hepatocytes, as described above. *N* = 5-6. All data are shown as the mean ± standard error of the mean. Comparisons were assessed by one-way ANOVA with Holm-Sidak post hoc tests (A-F). $*p < 0.05$, $**p < 0.01$, $***p < 0.001$. Scale bars, 100 μm.

Table 1

Primers for qPCR

	**Forward primer (5'-3')**	**Reverse primer (5'-3')**
18S	AGTCCCTGCCCTTTGTACACA	CGATCCGAGGGCCTCACTA
16S	TGATGCACTTGCAGAAAACA	ACCAGAGGAAATTTTCAATAGGC
B. adolescentis	GGTTTTCTGTGGCGCGGTTC	CTGGTGCCAAGGCATCCA
Cry1	CACTGGTTCCGAAAGGGACTC	CTGAAGCAAAAATCGCCACCT

Table 2

Key resources

**Antibodies**	**Sources**	**Identifier**
Cytochrome P450 1A1/2 Antibody	Affinity	Cat# AF5312
CYP2E1-specific polyclonal antibody	Proteintech	Cat# 19937-1-AP
PCNA antibody	Abmart	Cat# P60051
SAPK/JNK antibody	Cell Signaling Technology	Cat# 9252
Phospho-SAPK/JNK (Thr183/Tyr185) antibody	Cell Signaling Technology	Cat# 9255
Recombinant Anti - beta Actin antibody	Servicebio	Cat# GB15003
Recombinant Anti-GAPDH antibody	Servicebio	Cat# GB15004
Anti-rabbit IgG, HRP-linked Antibody	Cell Signaling Technology	Cat# 7074
HRP-conjugated Affinipure Goat Anti-Mouse IgG(H+L)	Proteintech	Cat# SA00001-1
TNF alpha Antibody	Affinity	Cat# AF7014
Anti-CD11b Rabbit pAb	Servicebio	Cat# GB115689

**Chemicals**	**Sources**	**Identifier**
Acetaminophen	Macklin	Cat# A800441
Hypaphorine	Macklin	Cat# H889706
KL001	Macklin	Cat# K921322
Acetaminophen sulfate potassium salt	Sigma	Cat# UC448
Acetaminophen glucuronide	Sigma	Cat# 43073

Sesame oil	ShanghaiyuanyeBio-Technology	Cat# S27343
de Man, Rogosa, and Sharpe broth	Hopebio	Cat# HB0384-5
Collagenase types IV	Worthington	Cat# LS004188
PBS	Gibco	Cat# C10010500BT
Collagen I	BD PharMingen	Cat# 354236
RPMI1640 medium	Gibco	Cat# C11875
Fetal Bovine Serum	Gibco	Cat# 10270-106
Penicillin/streptomycin	Gibco	Cat# 15140122
Bovine Serum Albumin	Sigma	Cat# V900933
Non-Fat Powdered Milk	Solarbio	Cat# D8340
TRIzol reagent	Invitrogen	Cat# 15596018
RIPA lysis buffer	Beyotime	Cat# P0013B
Primary Antibody Dilution Buffer	Beyotime	Cat# P0023A
Nitrocellulose membranes	Merck	Cat# HATF00010
Dihydroethidium	Thermo Scientific	Cat# D23107
Dichlorofluorescein diacetate (DCFH-DA) probe	Beyotime	Cat# S0033S
Mito-Tracker Red CMXRos	Beyotime	Cat# C1035
Hoechst 33258	Beyotime	Cat# C1011
ECL Western Blotting Substrate	Biosharp	Cat# BL520B
Multicolor Prestained Protein Ladder	Epizyme Biomedical Technology	Cat# WJ102
3,3'-Diaminobenzidine(DAB) Chromogenic Kit	ZSGB-BIO	Cat# ZLI-9018

**Commercial Assays**	**Sources**	**Identifier**
ALT assay kit	Nanjing Jiancheng Bioengineering Institute	Cat# C009-3-1

AST assay kit	Nanjing Jiancheng Bioengineering Institute	Cat# C010-2-1
SOD assay kit	Nanjing Jiancheng Bioengineering Institute	Cat# A001-3-2
CAT assay kit	Nanjing Jiancheng Bioengineering Institute	Cat# A007-1-1
GSH assay kit	Nanjing Jiancheng Bioengineering Institute	Cat# A006-2-1
T-GSH/GSSG assay kit	Nanjing Jiancheng Bioengineering Institute	Cat# A061-1-2
MDA assay kit	Beyotime	Cat# S0131S
NAPQI assay kit	Boshen	Cat# BS-E10517M1
CytoTox 96® Non-Radioactive Cytotoxicity Assay kit	Promega	Cat# G1780
Cell Counting kit-8	Meilunbio	Cat# MA0218
Mouse IL-6 ELISA kit	Neobioscience	Cat# EMC004
Mouse TNF-α ELISA kit	Neobioscience	Cat# EMC102a
Mouse MCP-1 ELISA kit	Neobioscience	Cat# EMC113
Mouse MCP-3 ELISA kit	Cusabio	Cat# CSB-E07426m
TUNEL Assay kit	KeyGEN	Cat# KGA7063
Reverse transcription reagent kit	Toyobo	Cat# FSQ-101
SYBR Green Master Mix	Toyobo	Cat# QPK-201C
BCA Protein Assay Kit	GLPBIO	Cat# GK10009
DNA extraction kit	Mabio	Cat# DNS362

**Software and algorithms**		
ImageJ software	NIH	https://imagej.nih.gov/ij/
R studio software	RStudio	https://www.rstudio.com/
Graphpad Prism 8 software	GraphPad	https://www.graphpad.com/scientific-software/prism/

Adobe illustrator 2021	Adobe	https://www.adobe.com/
Agilent LC1260 software	Agilent Technologies	https://www.agilent.com/
BioRender	BioRender	https://biorender.com/
STRING	STRING	https://string-db.org/
Cytoscape software	Cytoscape	https://cytoscape.org/
Gene Expression Omnibus database	GEO	https://www.ncbi.nlm.nih.gov/geo/
